# α-Linolenic Acid Alleviates Diabetic Cardiomyopathy by Activating AMPK-STAT3 Pathway to Inhibit Ferritinophagy and Enhance SLC7A11-GPX4 Antioxidant Axis

**DOI:** 10.3390/molecules31010079

**Published:** 2025-12-24

**Authors:** Ziqian Zhang, Xue Bai, Qian Du, Jianhong Yang

**Affiliations:** Medical School, University of Chinese Academy of Sciences, Beijing 101408, China; zhangziqian23@mails.ucas.ac.cn (Z.Z.); baixue22@mails.ucas.ac.cn (X.B.); duqian22@mails.ucas.ac.cn (Q.D.)

**Keywords:** diabetic cardiomyopathy, α-linolenic acid, ferroptosis, AMPK-STAT3 signaling pathway, ferritinophagy, SLC7A11/GSH/GPX4 axis

## Abstract

Diabetic cardiomyopathy (DCM) is a severe complication of diabetes, in which ferroptosis is a key pathogenic mechanism. This study examines how alpha-linolenic acid (ALA), a plant-derived omega-3 polyunsaturated fatty acid, protects against damage from ferroptosis in DCM. Using an in vitro model of H9C2 cardiomyocytes treated with high glucose/palmitate, combined with a high-fat diet and mouse model of low-dose streptozotocin (STZ)-induced diabetes, this research demonstrates for the first time that ALA significantly alleviates cardiac dysfunction and prevents ferroptosis. Mechanistically, ALA inhibits STAT3 phosphorylation by activating the AMPK signaling pathway, thereby reducing NCOA4-mediated ferritinophagy and mitigating mitochondrial iron overload and reactive oxygen species accumulation. It also enhances the function of the SLC7A11/GSH/GPX4 axis, reducing lipid peroxidation (LPO)-induced ferroptosis. Collectively, these findings indicate that ALA protects against diabetic cardiomyopathy by coordinating the regulation of ferritinophagy and antioxidant defense through the AMPK-STAT3 pathway, offering a potential therapeutic strategy for disease management.

## 1. Introduction

Diabetes mellitus (DM) is rapidly becoming a global concern, presenting a serious challenge to public health. The International Diabetes Federation (IDF) reports that around 589 million adults aged 20–79 will have diabetes in 2024, with projections indicating an increase to 853 million by 2050 [1]. Among the severe complications associated with DM, cardiovascular disease remains the primary contributor to mortality [2]. The cardiac implications of diabetes were first systematically described in 1972 by Rubler et al., who identified distinct pathological changes, including ventricular hypertrophy and fibrosis, in diabetic patients, coining the term diabetic cardiomyopathy (DCM) [3]. DCM is now considered a cardiac disorder specific to diabetes, marked by structural and functional impairments that occur independent of hypertension, coronary artery disease, valvular issues, or other heart conditions [4]. In DCM, an initial subclinical stage is present, featuring diastolic dysfunction that gradually evolves into systolic dysfunction, culminating in refractory heart failure with a lowered ejection fraction [5,6,7]. DCM is a critical driver underlying heart failure and mortality in patients with diabetes mellitus. However, its precise molecular mechanisms remain incompletely elucidated, and effective therapeutic strategies are currently lacking.

Ferroptosis is a unique form of regulated cell death, distinguished by the iron-dependent accumulation of lipid peroxides [8]. It has attracted significant interest due to its association with numerous pathological processes and is considered a promising therapeutic target [9]. Recent findings have confirmed that ferroptosis is a factor in the occurrence and development of cardiovascular diseases, including cardiomyopathy, myocardial infarction, myocardial ischemia–reperfusion injury, and heart failure [10,11,12,13,14]. Studies indicate that genes promoting ferroptosis were upregulated in the heart tissue of mice with STZ-induced diabetes [15], and ferroptosis was observed in mice with type 2 diabetes mellitus (T2DM) and DCM [16]. Studying heart tissue from diabetic patients with heart failure, Gawargi et al. [17] identified a gene profile that promotes ferroptosis. Inhibition of ferroptosis has been observed to improve heart contractile function in mice with T2DM [16]. Additionally, multiple experiments have confirmed that stopping ferroptosis helps reduce myocardial injury [18,19,20], effectively preserving cardiomyocytes, improving heart function [16,20], and delaying the progression of DCM. Ferroptosis inhibition is a new research direction for clinical intervention in DCM.

Autophagy, an evolutionarily conserved process for maintaining cellular homeostasis, promotes survival at basal levels but can induce autophagic cell death upon overactivation [21,22]. It is mechanistically linked to ferroptosis through the selective degradation of ferritin via the cargo receptor NCOA4, a process known as ferritinophagy [23,24,25,26,27,28]. Although NCOA4-mediated ferritinophagy supports iron bioavailability for mitochondrial function [29], dysregulated ferritinophagy results in pathological free iron overload (FIO), exacerbating oxidative stress via Fenton reactions and promoting ferroptosis [26,28,30]. In the heart, NCOA4-dependent ferritinophagy and cytosolic FIO activate the mitochondrial iron transporter SFXN1, culminating in mitochondrial iron accumulation and ferroptotic cell death in septic cardiomyopathy [31]. Cardiac-specific NCOA4 knockout or silencing attenuates ferritinophagy, ferroptosis, SFXN1-mediated mitochondrial FIO, and cardiomyocyte hypertrophy, thereby conferring protection against heart failure [32,33].

Ferroptosis is centrally regulated by the system Xc-/GSH/GPX4 axis, where System Xc- is an amino acid reverse transporter composed of SLC7A11 and SLC3A2 that can support glutathione synthesis. GPX4 utilizes GSH to reduce toxic lipid hydroperoxide (L-OOH) to non-toxic alcohols (L-OH), thereby suppressing ferroptosis [9,34,35,36]. Studies have demonstrated that the ferroptosis inhibitor Fer-1 protects against cardiomyocyte dysfunction induced by systemic Xc- deficiency [37]. Furthermore, hydrogen sulfide (H_2_S) sulfhydrates Keap1 to activate Nrf2 signaling, enhancing SLC7A11/GSH/GPX4 axis activity and protecting against doxorubicin-induced ferroptosis and myocardial injury [38,39,40].

ALA, a plant-derived n-3 polyunsaturated fatty acid (n-3 PUFA) abundant in nuts, leafy vegetables, and seed oils such as rapeseed, soybean, and flaxseed [41,42], is epidemiologically associated with reduced incidence of hypertension and is a potential adjuvant for cardiovascular prevention [43,44]. Mechanistically, ALA modulates lipid metabolism by inhibiting fatty acid synthesis and promoting mitochondrial β-oxidation, thereby lowering serum triglycerides, preventing intravascular lipid accumulation, and attenuating atherosclerosis [45]. It also exerts anti-inflammatory effects by reducing pro-inflammatory mediators, helping preserve endothelial function, and inhibiting plaque progression [46]. Experimental studies confirm that ALA-rich diets ameliorate cardiac oxidative stress and inflammation [47,48]; however, the molecular mechanisms underlying ALA’s cardioprotective effects, particularly its interplay with ferroptosis in the diabetic heart, remain incompletely understood.

In this study, we demonstrate in both a rat cardiomyocyte cell line and a mouse model that ALA activates AMPK phosphorylation while inhibiting STAT3 phosphorylation and nuclear translocation, thereby suppressing NCOA4-mediated ferritinophagy and attenuating mitochondrial reactive oxygen species production. Concurrently, ALA enhances the SLC7A11/GSH/GPX4 signaling axis by activating AMPK and inhibiting STAT3, ultimately reducing oxidative stress and ferroptosis and conferring protection against HG/PA-induced cardiomyocyte injury. These findings establish a mechanistic basis for the clinical application of ALA in treating diabetic cardiomyopathy associated with HG/PA conditions.

## 2. Results

### 2.1. ALA Attenuates HG/PA-Induced Ferroptosis in H9C2 Cells

**Figure 1:**
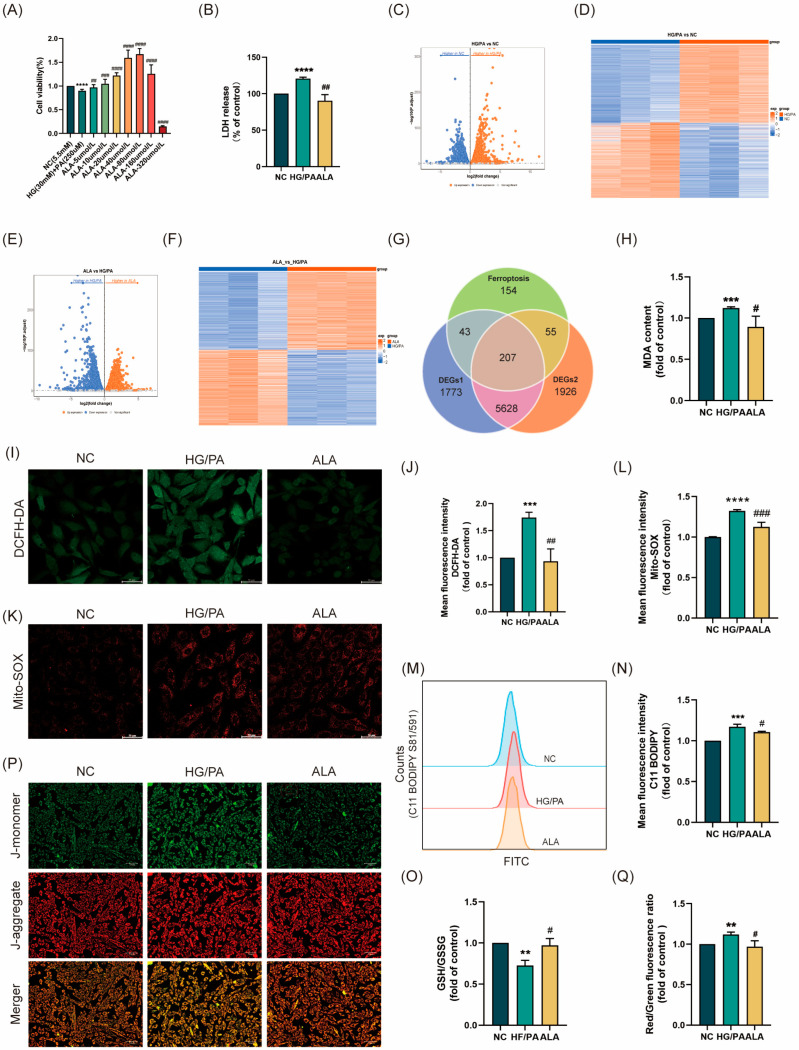
α-linolenic acid (ALA) attenuated HG/PA-induced ferroptosis in H9C2 cells. (**A**) Cell viability of HG/PA-injured H9C2 cells treated with different concentrations of ALA was determined by CCK-8 assay *(n* = 3). (**B**) LDH release in the culture medium (*n* = 3). (**C**,**D**) Volcano plot and heatmap showing differences between NC and HG/PA groups. (**E**,**F**) Volcano plot and heatmap differences between NC and HG/PA groups. (**G**) Venn diagram presenting the intersection between DEGs and ferroptosis regulators. (**H**) Measurement of malondialdehyde (MDA) levels (*n* = 3). (**I**,**J**) Representative images (scale bar = 50 μm, *n* = 3) and quantitative analysis of DCFH-DA staining for total intracellular ROS. (**K**,**L**) Representative images (scale bar = 50 μm, *n* = 3) and quantitative analysis of Mito-SOX staining for mitochondrial ROS. (**M**,**N**) Flow cytometry analysis of lipid peroxidation levels using C11-BODIPY 581/591 probe (*n* = 3). (**O**) Quantitative determination of intracellular GSH/GSSG ratio (*n* = 3). (**P**,**Q**) Representative images (scale bar = 100 μm, *n* = 3) and quantitative analysis of JC-1 staining (red: JC-1 aggregates; green: JC-1 monomers), reflecting mitochondrial membrane potential (ΔΨm) (*n* = 3). All results are presented as mean ± SD. ^#^ *p* < 0.05, **^/##^ *p* < 0.01, ***^/###^ *p* < 0.001, and ****^/####^ *p* < 0.0001 vs. the control group.

Firstly, based on preliminary laboratory research, we established a cardiomyocyte injury model using 30 mM of hyperglycemia (HG) and 250 μM of palmitate (PA). The cells were then treated with α-linolenic acid (ALA) at concentrations of 5, 10, 20, 40, 80, 160, and 320 μmol/L for 24 h. CCK-8 assay was performed to determine cell viability and identify the optimal therapeutic concentration of ALA. The results showed that 40 μM of ALA significantly improved cell viability in the HG/PA-injured cells compared to the model group (Figure 1A). To further investigate the protective effects of 40 μM of ALA against HG/PA-induced H9C2 cell injury, we measured lactate dehydrogenase (LDH) release in the culture medium. Compared with the NC group, HG/PA stimulation induced H9C2 cell injury, as evidenced by increased LDH release (Figure 1B), which was attenuated by ALA treatment. Next, we analyzed RNA sequencing data using the DESeq2 package (version 1.44.0) in R to compare gene expression levels between the HG/PA group and the NC group through differential analysis. Using P. adjust < 0.05 as the screening criterion, a total of 7651 differentially expressed genes (DEGs 1) were identified, including 3890 upregulated genes and 3761 downregulated genes (Figure 1C,D). A total of 7816 differentially expressed genes (DEGs 2) between the ALA group and the HG/PA group were identified, comprising 3965 upregulated genes and 3851 downregulated genes (Figure 1E,F). In the Venn diagram, the results of the two differential analyses were intersected with ferroptosis regulators. As shown in Figure 1G, 207 common differentially expressed genes were identified. Subsequently, to explore the protective role of ALA against HG/PA-induced ferroptosis in H9C2 cells, we determined the levels of the lipid peroxidation marker malondialdehyde (MDA), total reactive oxygen species (ROS), mitochondrial ROS (Mito-SOX), and lipid peroxidation-derived ROS. The results indicate that HG/PA treatment resulted in elevated levels of MDA (Figure 1H), total ROS (Figure 1I,J), mitochondrial ROS (Figure 1K,L), and lipid peroxidation-derived ROS (Figure 1M,N) and reduced glutathione (GSH) levels (Figure 1O), but these effects were mitigated by ALA administration. Abnormal changes in mitochondrial membrane potential (ΔΨm) are recognized as both a marker of mitochondrial dysfunction and an early indicator of ferroptosis [31]. HG/PA stimulation caused ΔΨm dissipation, as evidenced by a shift in JC-1 fluorescence from red (JC-1 aggregates) to green (JC-1 monomers), which was similarly reversed by ALA treatment (Figure 1P,Q). In conclusion, ferroptosis is involved in HG/PA-induced cardiomyocyte injury, and ALA effectively alleviates cardiomyocyte ferroptosis.

### 2.2. ALA Alleviates HG/PA-Induced Ferritinophagy in H9C2 Cells

**Figure 2:**
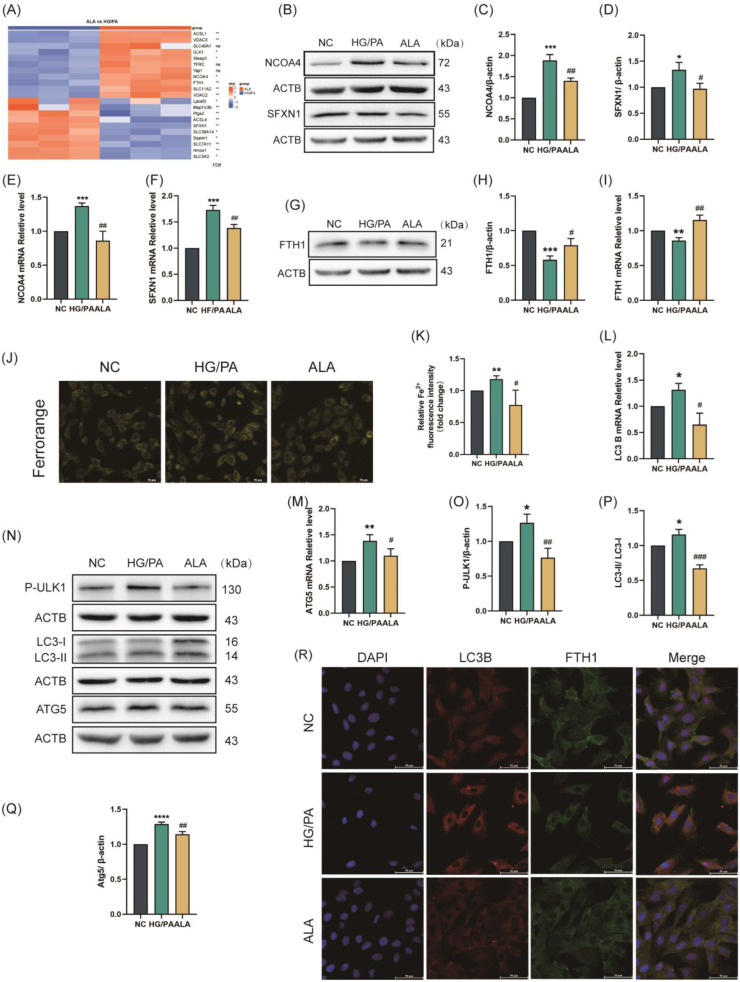
α-Linolenic acid (ALA) alleviated HG/PA-induced ferritinophagy in H9C2 cells. (**A**) Heatmap of significantly regulated genes associated with ferroptosis in ALA groups vs. HG/PA groups (*n* = 3). (**B**–**D**) Representative Western blot (**B**) and quantification analysis (**C**,**D**) of NCOA4 and SFXN1 protein levels in H9C2 (*n* = 3). (**E**,**F**) Relative mRNA levels of ferroptosis-related genes NCOA4 (**E**) and SFXN1 (**F**) detected by RT-qPCR (*n* = 3). (**G**,**H**) Representative Western blot (**G**) and quantitative analysis (**H**) of FTH1 protein expression (*n* = 3). (**I**) Relative mRNA level of ferroptosis-related gene FTH1 detected by RT-qPCR (*n* = 3). (**J**,**K**) Representative fluorescence images ((**J**), scale bar = 50 μm, *n* = 3) and quantitative analysis of fluorescence intensity (**K**) of intracellular Fe^2+^ in H9C2 cells (FerroOrange staining). (**L**,**M**) Relative mRNA levels of autophagy/ferroptosis-related genes LC3B (**L**) and ATG5 (**M**) detected by RT-qPCR (*n* = 3). (**N**–**Q**) Representative Western blot (**N**) and quantitative analysis (**O**–**Q**) of p-ULK1, LC3B, and ATG5 protein expression ((**O**): p-ULK1; (**P**): LC3B; (**Q**): ATG5, *n* = 3). (**R**) Immunofluorescence images showing colocalization of endogenous FTH1 (green) and LC3B (red) in H9C2 cells (scale bar = 50 μm); Nuclei were stained with DAPI (blue). Data are presented as means ± SDs from three independent experiments. *^/#^ *p* < 0.05, **^/##^ *p* < 0.01, ***^/###^ *p* < 0.001, and **** *p* < 0.0001, ^ns^ *p* > 0.05 vs. the indicated group.

Ferritinophagy is one of the canonical pathways regulating ferroptosis. During NCOA4-mediated ferritinophagy, ferritin degradation leads to elevated intracellular Fe^2+^ levels, which subsequently promote ferroptosis through the Fenton reaction. We discovered through gene heatmaps that the expression of ferroptosis-related genes in HG/PA and ALA groups and found that compared with cells in the HG/PA group, key ferritinophagy genes, including NCOA4, FTH1, and LC3B, were significantly upregulated in cells from the ALA group (Figure 1A). To investigate whether ALA regulates ferritinophagy, we assessed the mRNA and protein expression levels of the aforementioned key ferritin phagocytosis-associated factors. As illustrated in Figure 2B–F, HG/PA co-treatment significantly up-regulated both mRNA and protein levels of NCOA4 and SFXN1 compared to the NC group. Conversely, HG/PA exposure led to marked down-regulation of FTH1 at both transcriptional and protein levels (Figure 2G–I). Importantly, all these HG/PA-induced changes in NCOA4, SFXN1, and FTH1 expression were reversed by the ALA treatment. Consistent with the suppression of FTH1, a core iron storage protein, HG/PA stimulation significantly increased intracellular Fe^2+^ levels relative to the NC group. As anticipated, the ALA treatment effectively attenuated this Fe^2+^ accumulation (Figure 2J,K), coinciding with the restored expression of FTH1. We further investigated the effect of ALA on autophagic activation in HG/PA-injury cells by assessing autophagy-related markers. RT-qPCR analysis revealed that HG/PA co-treatment significantly elevated the mRNA levels of LC3B and ATG5 compared to in the NC cells (Figure 2L,M). Western blot results corroborated that HG/PA up-regulated the protein expression of p-ULK1, LC3B, and ATG5, all of which were suppressed by ALA administration (Figure 2N–Q). To visually evaluate ferritinophagy activation, we performed immunofluorescence staining to assess colocalization of endogenous FTH1 (green) and LC3B (red). As shown in Figure 2R, ALA treatment markedly reduced LC3B red fluorescence intensity, indicating attenuated autophagic activity, while increasing FTH1 green fluorescence, suggesting decreased ferritin degradation, relative to the HG/PA group. Together, these findings indicate that ALA effectively suppresses HG/PA-induced ferritinophagy in H9C2 cardiomyocytes.

### 2.3. ALA Ameliorates HG/PA-Induced Ferritinophagy in H9C2 by Activating the AMPK Signaling Pathway

**Figure 3:**
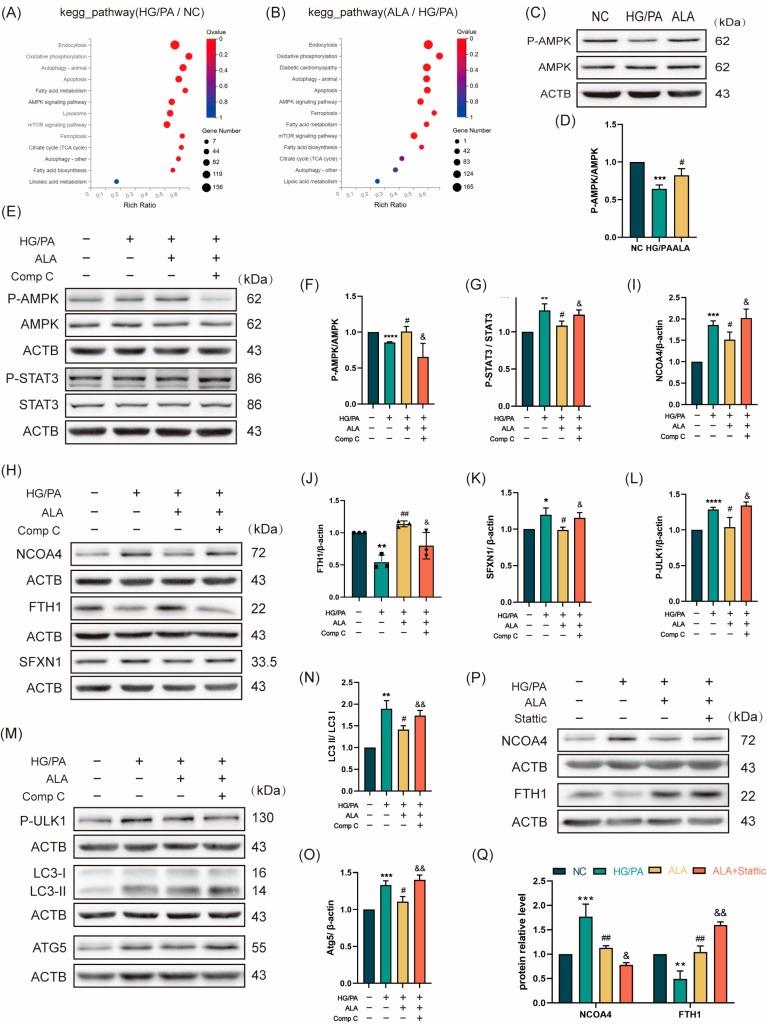
α-Linolenic acid (ALA) ameliorates HG/PA-induced ferritinophagy in H9C2 cells by activating the AMPK signaling pathway. (**A**) KEGG enrichment bubble plot comparing the NC group versus the HG/PA group (*n* = 3). (**B**) KEGG enrichment bubble plot comparing the ALA treatment group versus the HG/PA group (*n* = 3). (**C**,**D**) Representative Western blot and quantitative analysis of p-AMPK and AMPK protein expression in H9C2 cells (*n* = 3). (**E**–**G**) Representative Western blot (**E**) and quantification analysis (**F**,**G**) of p-AMPK, AMPK, p-STAT3, and STAT3 in H9C2 cells treated with HG/PA, ALA, and ALA+ Compound C (*n* = 3). (**H**–**K**) Representative Western blot (**H**) and quantification analysis (**I**–**K**) of NCOA4, FTH1, SFXN1 in H9C2 cells under different treatment conditions (*n* = 3). (**L**–**O**) Representative Western blot (**M**) and quantification analysis (**L**,**N**,**O**) of autophagy-related proteins (p-ULK1, LC3B, ATG5) in H9C2 cells after indicated treatments (*n* = 3). (**P**,**Q**) Representative Western blot (**P**) and quantification analysis (**Q**) of NCOA4, FTH1 in H9C2 cells treated with HG/PA, ALA, and ALA+ Stattic (5 μM, STAT3 phosphorylation inhibitor, *n* = 3). Note: Data is presented as mean ± SD from three independent experiments. *^/#/&^ *p* < 0.05, **^/##/&&^ *p* < 0.01, *** *p* < 0.001, and **** *p* < 0.0001.

To systematically investigate the mechanism by which ALA protects against high glucose/palmitic acid (HG/PA)-induced injury in H9C2 cardiomyocytes, we conducted a Kyoto Encyclopedia of Genes and Genomes (KEGG) enrichment analysis. The results revealed significant upregulation of the ferroptosis pathway in the HG/PA-treated cells compared to the NC group, which was effectively reversed by ALA treatment (Figure 3A,B). We next examined the involvement of the AMPK signaling pathway by assessing AMPK phosphorylation via Western blotting. ALA treatment significantly increased the p-AMPK/AMPK ratio under HG/PA conditions, indicating enhanced AMPK activation (Figure 3C,D). To determine whether ALA modulates P-STAT3/NCOA4/SFXN1-mediated ferritinophagy through AMPK activation, we inhibited AMPK using 5 μM of Compound C (Comp C), a specific pharmacological inhibitor. The cells were divided into four groups: NC, HG/PA, HG/PA + ALA, and HG/PA + ALA + Comp C. The Western blot analysis demonstrated that ALA increased the p-AMPK/AMPK ratio and decreased the p-STAT3/STAT3 ratio in the HG/PA-injured cells, suggesting AMPK activation and STAT3 inhibition. These effects were abolished by co-treatment with Comp C (Figure 3E–G). Furthermore, evaluation of ferritinophagy-related proteins (NCOA4, FTH1, SFXN1, p-ULK1, LC3B, and ATG5) showed that ALA downregulated the expression of NCOA4, SFXN1, p-ULK1, LC3B, and ATG5 while upregulating FTH1. These alterations were also reversed by Comp C (Figure 3H–O). Subsequently, we assessed the protein levels of NCOA4 and FTH1 following treatment with the STAT3 inhibitor Stattic. We found that ALA downregulated NCOA4 and FTH1 expression under HG/PA conditions, an effect further enhanced by co-treatment with Stattic. This suggests that ALA may mitigate ferritinophagy by regulating STAT3 (Figure 3P,Q). Together, these findings indicate that ALA attenuates HG/PA-induced ferritinophagy in H9C2 cells via by activating of the AMPK-STAT3 signaling pathway.

### 2.4. ALA Attenuates Ferroptotic Injury by Suppressing NCOA4-Dependent Ferritinophagy

**Figure 4:**
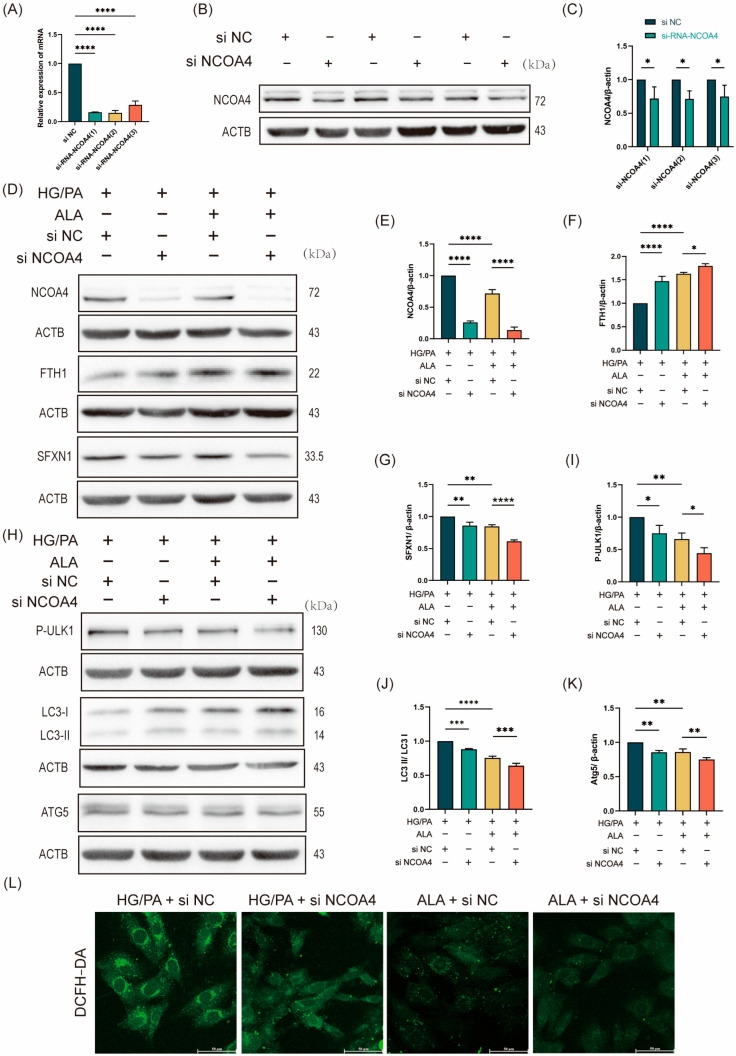
NCOA4-mediated ferritinophagy promotes ferroptosis, which is attenuated by ALA treatment. (**A**) RT-qPCR analysis of NCOA4 mRNA expression in H9C2 cells transfected with NCOA4-specific siRNAs (si NCOA4-1/2/3) or negative control siRNA (si NC) (*n* = 3). (**B**,**C**) Representative Western blot (**B**) and quantification analysis (**C**) of NCOA4 protein expression after siRNA transfection (*n* = 3). (**D**–**G**) Representative Western blot (**D**) and quantification analysis (**E**–**G**) of ferroptosis-related proteins (NCOA4, FTH1, SFXN1) in H9C2 cells under HG/PA conditions with or without ALA and si NCOA4 (*n* = 3). (**H**–**K**) Representative Western blot (**H**) and quantification analysis (**I**–**K**) of autophagy-related proteins (p-ULK1, LC3, ATG5) under the same experimental conditions (*n* = 3). (**L**) Intracellular ROS levels measured by DCFH-DA fluorescence staining (scale bar: 50 μm). Note: Data is expressed as mean ± SD from three independent experiments. * *p* < 0.05, ** *p* < 0.01, *** *p* < 0.001, and **** *p* < 0.0001 versus indicated group.

To investigate whether the protective effect of ALA against HG/PA-induced ferritinophagy is mediated through NCOA4, we performed siRNA-mediated knockdown of NCOA4 in H9C2 cells. Three distinct NCOA4-specific siRNAs were evaluated, all of which significantly reduced NCOA4 protein expression compared with the siRNA-negative control (si NC) group (Figure 4A–C). The most effective construction, si-RNA-NCOA4-1 (designated as si NCOA4), was selected for subsequent experiments. The cells were then divided into four treatment groups: HG/PA + si NC, HG/PA + si NCOA4, HG/PA + ALA + si NC, and HG/PA + ALA + si NCOA4. Western blot analysis of ferroptosis-related proteins demonstrated that NCOA4 knockdown alone decreased NCOA4 and SFXN1 expression while increasing FTH1 levels under HG/PA conditions. Similarly, ALA treatment reduced NCOA4 and SFXN1 and elevated FTH1 expression. Notably, the combination of ALA and si NCOA4 resulted in more pronounced suppression of NCOA4 and SFXN1 and stronger upregulation of FTH1 (Figure 4D–G). We then examined the expression of autophagy-related proteins. Consistent with the above findings, both NCOA4 knockdown and ALA treatment individually reduced the levels of p-ULK1, LC3B, and ATG5 in the HG/PA-injured cells. Moreover, their combination synergistically enhanced the downregulation of these autophagic markers (Figure 4H–K). To further evaluate the functional impact of NCOA4 inhibition, intracellular ROS levels were measured using DCFH-DA staining. NCOA4 knockdown significantly attenuated HG/PA-induced ROS accumulation. Furthermore, ALA co-treatment further potentiated this antioxidant effect (Figure 4L). Collectively, these results indicate that ALA attenuates ferroptosis in HG/PA-stimulated H9C2 cells, primarily by inhibiting NCOA4-mediated ferritinophagy.

### 2.5. ALA Mitigates Ferroptosis in H9C2 by Inhibiting STAT3 Phosphorylation and Activating the SLC7A11/GSH/GPX4 Antioxidant Axis

**Figure 5:**
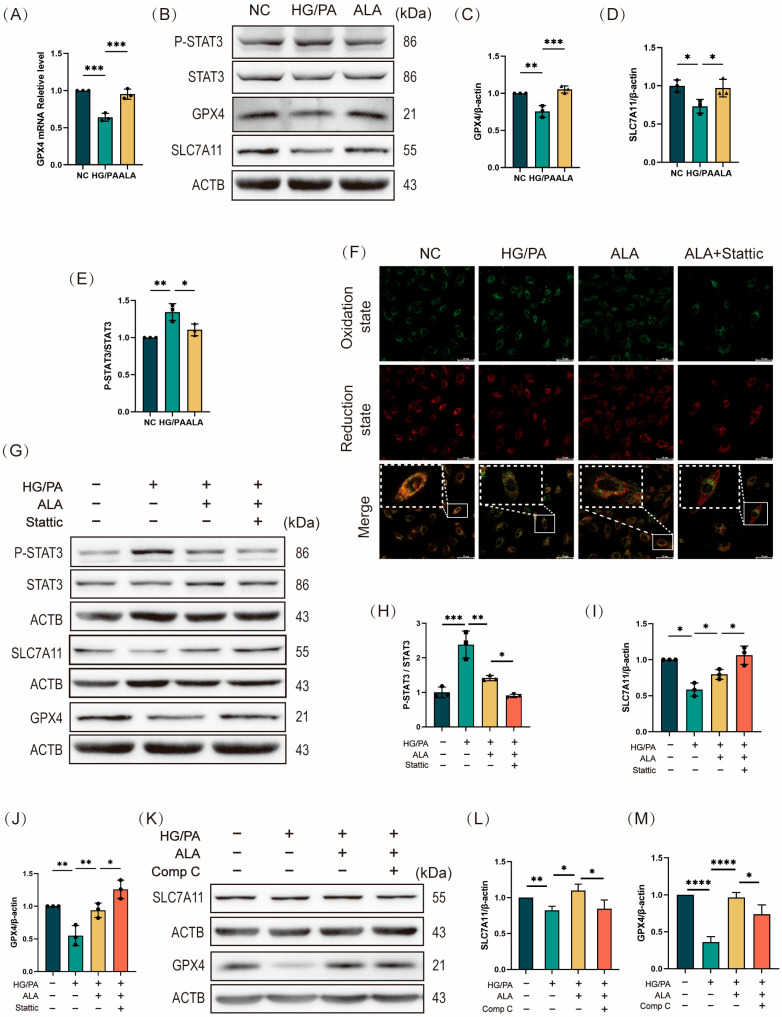
α-Linolenic acid (ALA) alleviates myocardial ferroptosis by inhibiting STAT3 phosphorylation to promote the activation of the SLC7A11/GSH/GPX4 axis. (**A**) Relative mRNA expression level of GPX4 (detected by reverse transcription-quantitative polymerase chain reaction, RT-qPCR) in H9C2 cardiomyocytes (*n* = 3). (**B**–**E**) Representative Western blot (**B**) and quantification analysis (**C**–**E**) of p-STAT3/STAT3 ratio, GPX4, and SLC7A11 (*n* = 3). (**F**) Representative fluorescence images of lipid peroxides in H9C2 cells treated with HG/PA, ALA, or ALA + Stattic (5 μM, STAT3 phosphorylation inhibitor), detected by BODIPY 581/591 C11 staining. Green indicates the oxidized state, while red indicates the reduced state. (scale bar: 50 μm). (**G**–**J**) Representative Western blot (**G**) and quantification analysis (**H**–**J**) of p-STAT3, total STAT3, GPX4, and SLC7A11 in H9C2 cells treated with HG/PA, ALA, or ALA + Stattic (*n* = 3). (**K**–**M**) Representative Western blot (**K**) and quantification analysis (**L**,**M**) of SLC7A11, GPX4 in H9C2 cells treated with HG/PA, ALA, and ALA+ Comp C (*n* = 3). All results are expressed as mean ± SD. * *p* < 0.05, ** *p* < 0.01, *** *p* < 0.001, and **** *p* < 0.0001.

Given that lipid peroxidation is a central hallmark of ferroptosis, we next investigated the mechanism by which ALA inhibits LPO-induced ferroptosis, focusing on the SLC7A11/GSH/GPX4 axis—a key antioxidant pathway that antagonizes ferroptosis. GPX4 acts as a critical regulator of LPO by utilizing reduced glutathione (GSH) to reduce toxic lipid hydroperoxides to non-toxic alcohols, thereby preventing lipid peroxidation-driven cell death. Compared to the NC group, HG/PA co-treatment significantly downregulated GPX4 mRNA expression in H9C2 cells. ALA treatment markedly reversed this downregulation, increasing GPX4 expression at both transcriptional and translational levels (Figure 5A–C), suggesting that ALA enhances GPX4-mediated LPO clearance. Since GPX4 activity depends on intracellular GSH availability, we measured GSH levels across treatment groups. Consistent with earlier findings (Figure 1L), HG/PA exposure substantially decreased intracellular GSH compared to NC, while ALA restored GSH to near-normal levels. GSH biosynthesis depends on cystine uptake via SLC7A11, a cystine/glutamate antiporter. Western blot analysis confirmed that ALA reversed the HG/PA-induced downregulation of SLC7A11 protein expression (Figure 5B,D), indicating that ALA facilitates GSH synthesis by promoting SLC7A11-mediated cystine uptake. To further investigate the role of STAT3, a transcription factor involved in regulating ferroptosis (Figure 5B,E), in the protective effects of ALA, we employed Stattic to selectively inhibit STAT3 phosphorylation by targeting Tyr750 phosphorylation. H9C2 cells were divided into four groups: NC, HG/PA, HG/PA + ALA, and HG/PA + ALA + Stattic (5 μM; 2 h pre-treatment). Lipid ROS levels were assessed using a BODIPY 581/591 C11 probe. HG/PA stimulation markedly increased lipid ROS fluorescence, indicating LPO accumulation, while ALA significantly attenuated this effect. Co-treatment with Stattic further reduced lipid ROS, suggesting that STAT3 inhibition synergizes with ALA to suppress LPO (Figure 5F). We then evaluated the protein levels of p-STAT3, total STAT3, SLC7A11, and GPX4 across groups. HG/PA increased the p-STAT3/STAT3 ratio, indicating STAT3 activation, while ALA reduced it. Stattic enhanced ALA-induced STAT3 dephosphorylation (Figure 5G,H). Concurrently, ALA upregulated SLC7A11 and GPX4 expression under HG/PA conditions, an effect further amplified by Stattic co-treatment (Figure 5G,I,J). Finally, we also examined the protein levels of SLC7A11 and GPX4 following treatment with the AMPK inhibitor Comp C. We found that ALA upregulates SLC7A11 and GPX4 expression under HG/PA conditions, an effect that is reversed upon addition of Comp C. This indicates that the SLC7A11/GPX4 antioxidant axis is regulated by AMPK (Figure 5K–M). In summary, ALA may promote the expression of the SLC7A11/GSH/GPX4 pathway by inhibiting STAT3 phosphorylation, thereby enhancing the antioxidant capacity of H9C2 cardiomyocytes and attenuating HG/PA-induced iron overload, ultimately reducing LPO accumulation.

### 2.6. ALA Ameliorates Cardiac Dysfunction in Mice with Diabetes Induced by High-Fat Diet and Low-Dose Streptozotocin

**Figure 6:**
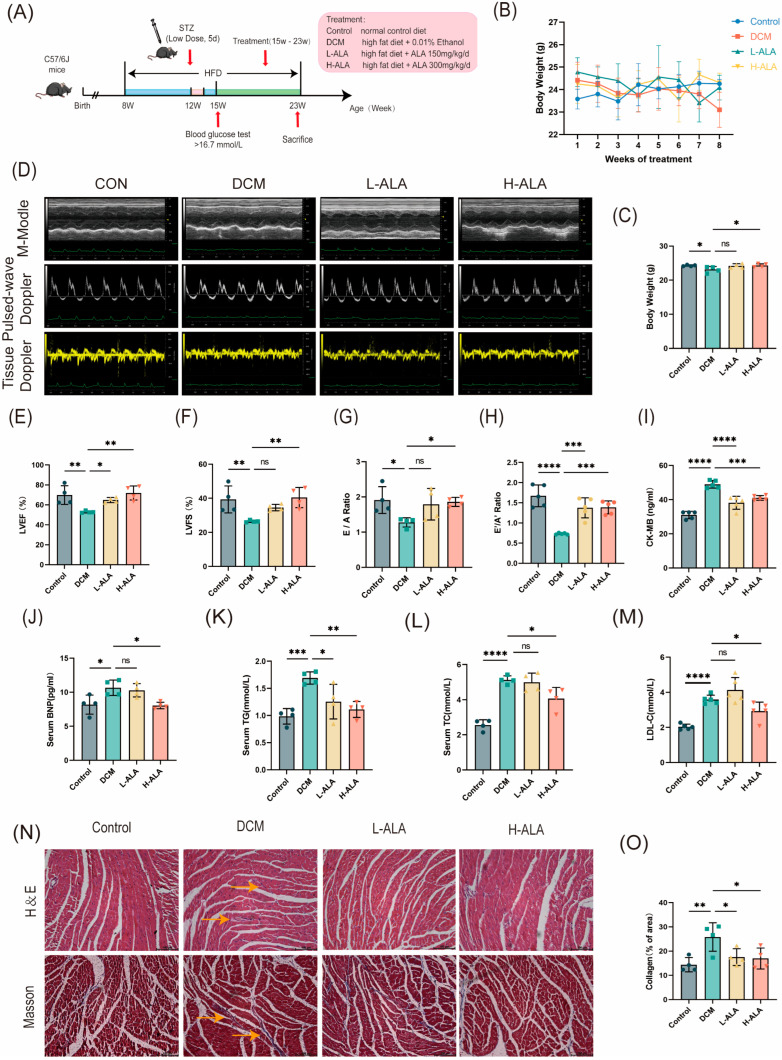
α-Linolenic Acid (ALA) alleviates cardiac dysfunction in mice with diabetes induced by HFD combined with low-dose STZ. (**A**) Animal protocol. (**B**) Body weight change curves recorded during ALA administration period (*n* = 4). (**C**) Body weight (*n* = 4). (**D**) Representative echocardiography images for cardiac function assessment. (**E**,**F**) Left ventricular ejection fraction (LVEF%, C, *n* = 4) and left ventricular fractional shortening (LVFS%, D, *n* = 4). (**G**,**H**) Diastolic function indices: E/A ratio ((**G**), *n* = 4); E’/A’ ratio ((**H**), *n* = 5). (**I**–**M**) Serum levels of creatine kinase–MB (CK–MB, (**I**), *n* = 5), brain natriuretic peptide (BNP, (**J**), *n* = 4), triglycerides (TG, (**K**), *n* = 4), total cholesterol (TC, (**L**), *n* = 4), and low-density lipoprotein cholesterol (LDL–C, (**M**), *n* = 5). (**N**) Representative hematoxylin and eosin (H&E) and Masson’s trichrome staining of myocardial tissue (Yellow arrows indicate tissue infiltration and fibrosis, scale bar: 100 μm). (**O**) Quantitative analysis of myocardial fibrosis (*n* = 4). All results are expressed as mean ± SD. * *p* < 0.05, ** *p* < 0.01, *** *p* < 0.001, and **** *p* < 0.0001, ^ns^ *p* > 0.05.

Eight-week-old healthy C57BL/6J mice were randomly allocated into four groups. Mice fed a normal diet served as the control group. Type 2 diabetes was induced with a high-fat diet combined with a low-dose streptozotocin (STZ) injection. Mice in which diabetes was successfully induced were then randomly divided into three groups based on their average body weight: diabetic cardiomyopathy model group (DCM), a low-dose ALA treatment group (150 mg/kg/day; L-ALA), and a high-dose ALA treatment group (300 mg/kg/day; H–ALA). The treatment lasted 8 weeks (Figure 6A). During the ALA treatment, the mice in the ALA group gradually gained weight (Figure 6B). Body weight decreased in the DCM model group and increased in the high-dose ALA group, and no significant difference was found between the low-dose group and the DCM group (Figure 6C). After the treatment period, cardiac structure and function were evaluated using echocardiography and Doppler ultrasound. Compared with the control group, the diabetic mice exhibited impaired cardiac systolic function (with preserved ejection fraction). High-dose ALA treatment significantly improved left ventricular ejection fraction (LVEF%) and left ventricular fractional shortening (LVFS%), but no significant improvement was observed in the low-dose group (Figure 6D–F). The DCM group showed decreased E/A and E′/A′ ratios, which increased after high-dose ALA treatment (Figure 6G,H). Serum levels of creatine kinase-MB (CK–MB), brain natriuretic peptide (BNP), total cholesterol (TC), triglycerides (TG), and low-density lipoprotein cholesterol (LDL–C) were significantly elevated in the diabetic mice compared to the controls. High-dose ALA treatment markedly reduced these biomarkers, but no significant changes were observed in the low-dose group (Figure 6I–M). H&E staining revealed well-organized, densely arranged cardiomyocytes with intact fibers in the control mice. In contrast, the cardiomyocytes in the DCM group exhibited disordered cell arrangement, irregular morphology, and localized fiber disruption. High-dose ALA treatment improved myocardial cell alignment and reduced fiber breakage, with better structural preservation than in the low-dose group (Figure 6N). Masson staining indicated that ALA treatment significantly attenuated myocardial fibrosis in the DCM mice (Figure 6N,O). These results demonstrate that high-dose α-linolenic acid alleviates cardiac dysfunction in mice with diabetes induced using a high-fat diet and low-dose streptozotocin.

### 2.7. ALA Inhibits Ferroptosis in Mice with Diabetes Induced by HFD Combined with Low-Dose STZ

**Figure 7:**
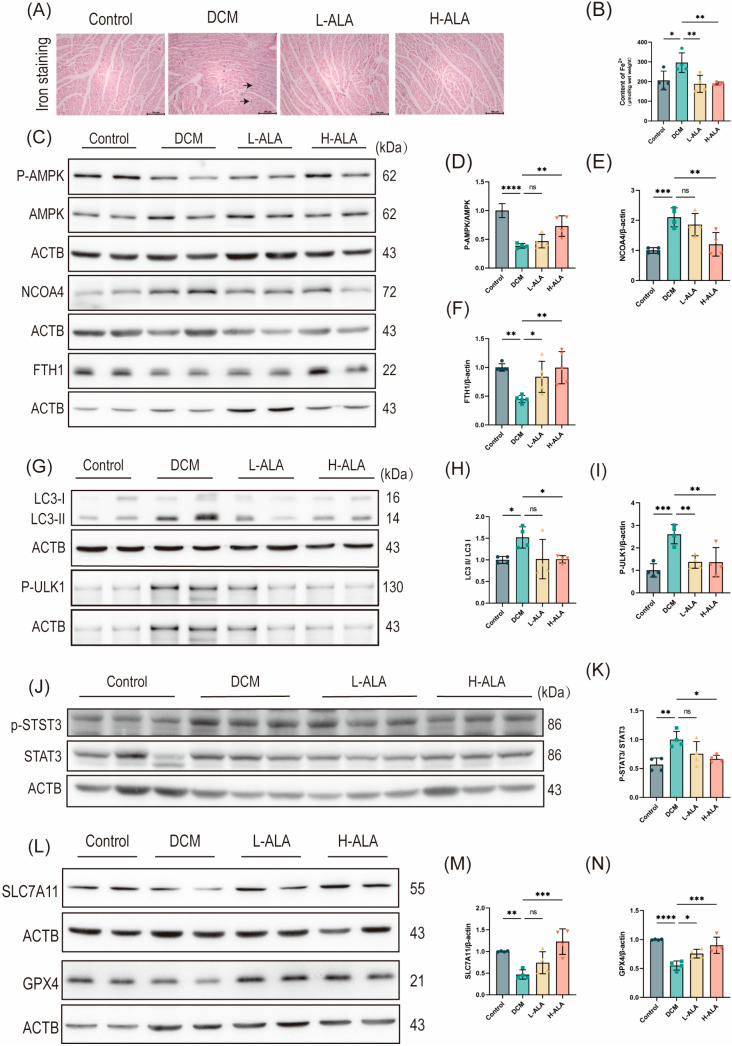
High-dose ALA treatment inhibits ferritinophagy in mice with diabetes induced by high-fat diet and low-dose STZ. (**A**) Representative images of Prussian blue staining, black arrows represent the sites of iron ion stasis. (scale bare: 100 μm). (**B**) Intracellular iron ion content in cardiac tissues (*n* = 4). (**C**–**F**) Representative Western blot (**C**) and quantification analysis (**D**–**F**) of p-AMPK, AMPK, NCOA4, and FTH1 in myocardial tissues (*n* = 4). (**G**–**I**) Representative Western blot (**G**) and quantification analysis (**H**,**I**) of autophagy-related proteins LC3B and p-ULK1 (*n* = 4). (**J**,**K**) Representative Western blot (**J**) and quantification analysis (**K**) of p-STAT3, STAT3 protein expression (*n* = 4). (**L**–**N**) Representative Western blot (**L**) and quantification analysis (**M**,**N**) of SLC7A11 and GPX4 protein expression (*n* = 4). Note: Data are presented as means ± SDs. * *p* < 0.05, ** *p* < 0.01, *** *p* < 0.001, and **** *p* < 0.0001, ^ns^ *p* > 0.05.

We further evaluated the role of ferroptosis in HFD + low-dose STZ-induced diabetes in mice. Prussian blue staining showed that HFD + low-dose STZ treatment significantly increased iron accumulation in cardiac tissues (Figure 7A,B). To further validate the mechanism by which ALA alleviates DCM at the animal level, we collected mouse heart tissues and determined the expression of proteins related to the AMPK metabolic pathway. Compared with the control group, the phosphorylation level of AMPK was decreased in the DCM group; this reduction was reversed to normal levels following high-dose ALA treatment (Figure 7C,D). The DCM group also exhibited significantly higher protein levels of NCOA4, LC3B, and p-ULK1 and a lower FTH1 protein level compared with the control group. High-dose ALA treatment downregulated NCOA4, LC3B, and p-ULK1 while upregulating FTH1. Low-dose ALA had no significant effect on these proteins (Figure 7C,E–I), suggesting that ALA may alleviate DCM-induced ferritinophagy by activating the AMPK signaling pathway and inhibiting the expression of NCOA4 and SFXN1. Finally, we detected the protein expression of p-STAT3, STAT3, SLC7A11 and GPX4 in cardiac tissues. Following HFD + low-dose STZ treatment, the p-STAT3/STAT3 ratio increased. ALA treatment downregulates STAT3 phosphorylation (Figure 7J,K). The DCM group showed decreased levels of SLC7A11 and GPX4, but high-dose ALA treatment significantly increased their expression (Figure 7L–N). These findings demonstrate that ALA treatment mitigates oxidative stress-induced ferroptosis in mice with diabetes induced by an HFD combined with low-dose STZ, activating the SLC7A11/GPX4 axis, a core antioxidant mechanism that counteracts lipid peroxidation. Collectively, high-dose ALA suppresses ferritinophagy and activates the SLC7A11/GSH/GPX4 antioxidant axis via the AMPK-STAT3 signaling pathway to alleviate ferroptosis-induced diabetic cardiomyopathy.

## 3. Discussion

**Figure 8:**
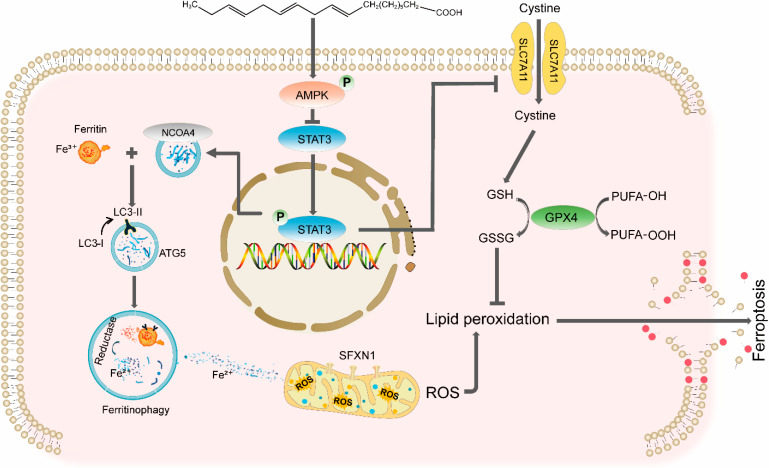
Schematic diagram of ALA alleviating ferroptosis in diabetic cardiomyopathy.

DCM remains a critical complication of diabetes mellitus, characterized by progressive cardiac structural and functional impairment independent of hypertension or coronary artery disease. Despite advances in understanding its pathogenesis, targeted therapies are lacking. Here, we demonstrate that ALA, a plant-derived ω-3 polyunsaturated fatty acid, alleviates DCM by suppressing ferroptosis through two mechanisms: inhibiting NCOA4-mediated ferritinophagy and activating the SLC7A11/GSH/GPX4 antioxidant axis, both of which are regulated by the AMPK-STAT3 signaling pathway (Figure 8). These findings provide novel insights into the interplay between iron metabolism, redox balance, and metabolic signaling in DCM.

Based on autopsy findings, Rubler et al. [3] defined diabetic cardiomyopathy (DCM) as a distinct ventricular structural and functional abnormality in diabetic patients that occurs independent of hypertension, coronary artery disease, or other known cardiac complications. In recent years, multiple mechanisms have been proposed to contribute to this clinical condition, including oxidative stress, fibrotic processes, cardiomyocyte death, mitochondrial dysfunction, and alterations in myocardial energy metabolism [38,49,50]. Nevertheless, the exact etiology of DCM remains unclear, and no specific treatment is currently available. To investigate the pathogenic mechanisms underlying DCM and evaluate the therapeutic potential of ALA, we employed both cellular and in vivo models. In the in vitro model, H9C2 cells were treated with high glucose and HG/PA to mimic diabetic cardiomyopathy. Successful induction of the DCM phenotype was confirmed by reduced cell viability, increased LDH release, excessive generation of ROS and MDA, intracellular lipid peroxidation, and loss of mitochondrial membrane potential (Figure 1). An in vivo model of DCM was established in C57BL/6J wild-type male mice by feeding them a high-fat diet for four weeks, followed by low-dose STZ administration for five consecutive days. Successful model establishment was evidenced by impaired cardiac function, structural abnormalities in cardiac tissue, dyslipidemia, and elevated serum biomarkers associated with cardiac dysfunction (Figure 6).

ALA is abundant in nuts, leafy vegetables, and plant oils such as canola, soybean, and flaxseed oil [41,42]. ALA has been shown to modulate lipid metabolism. It can significantly reduce serum triglyceride levels by inhibiting enzymes involved in fatty acid synthesis and enhancing mitochondrial β-oxidation [45]. This effect helps prevent lipid accumulation in the bloodstream, a key factor in the development of atherosclerosis. ALA also exhibits anti-inflammatory properties. Chronic inflammation is a crucial contributor to the pathogenesis of cardiovascular diseases. By competing with arachidonic acid for metabolic enzymes, ALA can reduce the production of pro-inflammatory mediators [46]. This anti-inflammatory effect helps maintain vascular endothelial function and prevent the accumulation of atherosclerotic plaque. While accumulating evidence has established ALA’s cardioprotective effects, its underlying mechanisms remain poorly understood. In this study, we elucidated a novel regulatory mechanism by which ALA exerts protective effects against DCM.

Ferroptosis is a unique type of regulated cell death (RCD) driven by iron-dependent lipid peroxidation, which is closely associated with the onset and progression of cardiovascular diseases, including cardiomyopathy, heart transplantation-associated pathologies, vascular injury, stroke, myocardial ischemia/reperfusion injury, and heart failure [38]. To date, only a handful of recent studies have reported a close association between ferroptosis and DCM initiation [16,51]. For instance, Wang and colleagues demonstrated that ferroptosis occurs in the myocardium of type 2 diabetic mice, and its inhibition via liproxstatin-1 mitigates the progression of diastolic dysfunction [52]. In diabetes, increased advanced glycation end products [16], lipid peroxidation, and oxidative stress [53] contribute to the pathogenesis of DCM; these factors also trigger cellular iron overload and ferroptosis. The expression of ferroptosis-promoting genes was found to be increased in the heart tissue of STZ-induced diabetic mice [15], and ferroptosis was observed in T2DM mice with DCM [16]. Consistent with these observations, our results demonstrate that both HG/PA stimulation in H9C2 cardiomyocytes and the induction of diabetes in mice via an HFD combined with low-dose STZ trigger hallmark features of ferroptosis. These features include elevated intracellular and cardiac tissue iron levels, as demonstrated by FerroOrange and Prussian blue staining; increased ROS, as detected by DCFH-DA and Mito-SOX; elevated MDA levels; enhanced lipid peroxidation, as assessed using BODIPY 581/591 C11; and depleted GSH and mitochondrial dysfunction, as monitored using JC-1. Importantly, our research has, for the first time, found that ALA is able to reverse these ferroptotic changes, thereby confirming its potential as a ferroptosis inhibitor in the context of DCM.

Ferritinophagy, the selective autophagic degradation of ferritin by NCOA4, is a critical upstream regulator of ferroptosis, as it releases labile iron to fuel lipid peroxidation. Ferritinophagy-induced cell death has been implicated in cigarette smoke-induced chronic obstructive pulmonary disease (COPD) [54], apelin-13-induced cardiomyocyte hypertrophy [55], sepsis-induced cardiac injury [31], and zinc oxide nanoparticle-induced endothelial dysfunction [56]. However, to date, no studies have examined the role of ALA in regulating NCOA4-mediated ferritinophagy in the context of cardiac injury associated with diabetic cardiomyopathy. Our experiments demonstrate both in vivo and in vitro that HG/PA administration induces ferroptosis through ferritinophagy, leading to DCM. Our study is also the first to show that ALA suppresses this process in DCM. HG/PA and HFD/STZ upregulated NCOA4 and SFXN1 while downregulating FTH1, leading to iron overload. ALA reversed these trends, reducing NCOA4/SFXN1 and increasing FTH1. Additionally, ALA reduced autophagy markers (p-ULK1, LC3B, and ATG5) and disrupted FTH1-LC3B colocalization, confirming inhibited ferritinophagy. The results demonstrate that inhibition of NCOA4 can attenuate HG/PA-induced increases in ROS levels. This inhibitory effect also affected the expression of related proteins, including SFXN1, FTH1, LC3B, and ATG5. Thus, ALA’s inhibition of NCOA4-ferritinophagy is a novel means of limiting iron-dependent cell death in DCM.

The SLC7A11/GSH/GPX4 axis is a core antioxidant pathway that counteracts lipid peroxidation and inhibits ferroptosis. SLC7A11, a critical component of the system Xc complex, mitigates iron overload-induced ferroptosis by regulating cystine uptake [39]. SLC7A11 imports cystine for GSH synthesis, while GPX4 uses GSH to detoxify lipid hydroperoxides. Recent publications suggest that SLC7A11 prevents transverse aortic constriction (TAC)-induced cardiac remodeling and iron overload cardiomyopathy by suppressing ferroptosis [57,58]. Additionally, research has demonstrated that SLC7A11 suppresses pathological hypertrophy by blocking Ang II-induced ferroptosis [37]. Our data show that HG/PA and HFD/STZ downregulated SLC7A11 and GPX4, reducing GSH and impairing lipid peroxide clearance. Thus, ALA’s modulation of the SLC7A11/GSH/GPX4 axis may represent a context-specific strategy to enhance antioxidant defenses in DCM.

Our study reveals that ALA effectively inhibits NCOA4-mediated ferritinophagy in DCM, as evidenced by downregulation of NCOA4 and autophagy-related proteins, and by the restoration of FTH1 levels. Interestingly, our NCOA4 knockdown experiments provide deeper insights into ALA’s mechanism of action. We observed that when ALA treatment was combined with NCOA4 silencing, it had an additive or even synergistic protective effect (Figure 4). Jia et al. (2009) [59] reported that such synergism typically indicates that combined interventions may target parallel or independent pathways. This strongly suggests that while NCOA4-mediated ferritinophagy is a key pathway regulated by ALA, it is not the sole mechanism underlying its potent anti-ferroptotic effects. This interpretation was further corroborated by our subsequent finding, illustrated in Figure 5, that ALA also potently activates the classical SLC7A11/GSH/GPX4 antioxidant axis. Thus, a more comprehensive explanation emerges: ALA exerts its cardioprotective effects through a dual mechanism: reducing the supply of unstable iron by inhibiting ferritinophagy and enhancing the cellular ‘defense’ system for clearing lipid peroxides. This multi-target strategy, which simultaneously affects iron availability and lipid peroxide clearance, likely explains ALA’s remarkable efficacy against ferroptosis in DCM, and is a newly discovered aspect of its biological activity.

AMPK mitigates myocardial remodeling and fibrosis by suppressing excessive autophagy and correcting lipid metabolism disorders in cardiomyopathy, while STAT3, which binds to promoters of ferroptosis-related genes such as SLC7A11 and GPX4, can promote ferroptosis and exacerbate pathology in non-tumor contexts [60]. Notably, AMPK inhibits STAT3 transcriptional activity via phosphorylation at Ser727, thereby reducing cytokine release and alleviating myocardial inflammation [61]. Our data shows that ALA activated AMPK while inhibiting STAT3 phosphorylation in both HG/PA-treated H9C2 cells and mice with STZ-induced diabetes. Pharmacological interventions confirmed this crosstalk: the AMPK inhibitor Compound C abolished ALA-mediated STAT3 dephosphorylation, suppression of ferritinophagy, and upregulation of the SLC7A11/GPX4 axis, whereas the STAT3 inhibitor Stattic enhanced ALA’s ability to reduce lipid ROS and elevate SLC7A11 and GPX4 expression. This aligns with reports that STAT3 represses SLC7A11/GPX4 in non-malignant settings but promotes ferroptosis resistance in cancer [60]. These results demonstrate that AMPK activation controls the ferroptosis pathway by inhibiting STAT3. This extends previous findings that AMPK suppresses ferroptosis in DCM via lipid metabolic and mitochondrial regulation [16,62], and identifies STAT3 inhibition as a novel downstream mechanism through which AMPK alleviates ferroptosis. In summary, our study highlights the AMPK–STAT3 axis as a central regulator of ferroptosis in DCM, whereby ALA may be activate through AMPK to inhibit STAT3, thereby concurrently suppressing ferritinophagy and enhancing antioxidant defenses. These findings underscore the therapeutic potential of targeting this pathway.

## 4. Materials and Methods

### 4.1. Animals and Animal Models

Male C57BL/6J mice (8 weeks old) were obtained from Beijing Langke Biotechnology Co., Ltd. (Beijing, China). The mice were randomly divided into a control group and a diabetic cardiomyopathy (DCM) model group. The DCM group was fed a high-fat diet (HFD; 60% fat, 20% protein, 20% carbohydrates; XIAOSHUYOUTAI, Beijing, China) for 4 weeks, followed by intraperitoneal (i.p.) injections of streptozotocin (STZ, S1030, Sigma-Aldrich, St. Louis, MI, USA; formulated in 0.1 M citrate buffer; pH = 4.5) for 5 consecutive days to induce T2DM [63]. T2DM induction was confirmed 2 weeks after STZ administration based on random blood glucose (RBG) levels > 16.7 mmol/L. The control mice received citrate buffer vehicle injections on the same schedule. Following successful T2DM induction, the diabetic mice were randomized into three groups. A model control group received the vehicle (0.01% ethanol) via oral gavage, the low-dose α-linolenic acid treatment group (ALA, GC19540, GLPBIO, Central Ave, Montclair, CA, USA) received 150 mg/kg/day, and the high-dose ALA treatment group received 300 mg/kg/day. ALA (>99.50% purity) was dissolved in 0.01% ethanol and administered for 8 weeks. Following echocardiography, the mice were anesthetized with isoflurane, followed by plasma extraction (the mice were fasted for 6 h prior to plasma extraction) and euthanasia by spinal dislocation. For the entire duration of the experiment, the rodents were kept in a 24 °C ± 1 °C temperature-controlled habitat, exposed to a 12-h light/12-h dark cycle, placed 5 cages of mice in each cage, and had unrestricted access to both food and water.

### 4.2. Cell Culture and Treatment

The H9C2 embryonic rat heart-derived cell line was purchased from Procell (CL-0089, Wuhan, China) and cultured in Dulbecco’s modified Eagle medium (DMEM, Gibco, Thermo Fisher Scientific, Inc., Waltham, MA, USA) containing 10% fetal bovine serum (FBS, C04001-500, Viva Cell BIOSCIENCES, XP, BioMed, Shanghai, China) and 1% penicillin streptomycin (C3420-0100, Hy Clone, South Logan, UT, USA) at 37 °C with 5% CO_2_.

Based on previous experiments conducted in our laboratory [64], we used a 30 mmol/L of glucose (Macklin Biochemical Technology Co., Ltd., Shanghai, China) and 250 μM of palmitic acid (PA) treatment, administered for 24 h, to establish the model group (HG/PA). In preliminary in vitro experiments, varying concentrations of ALA (10, 20, 40, 80, 160, and 320 μmol/L, L105575, Aladdin Biochemical Technology Co., Ltd., Shanghai, China) were added to the cells cultured under 30 mmol/L of glucose and 250 μM of PA (HG/PA). Finally, we selected the 40 μmol/L of ALA treatment. In further experiments, H9C2 cells were treated 24 h prior to collecting with either 5.5 mmol/L of glucose (NC group), 30 mmol/L of glucose plus 250 μM of PA (HG/PA group), or 30 mmol/L of glucose plus 250 μM of PA plus 40 μmol/L of ALA (ALA group). In a third set of in vitro experiments, H9C2cells were pre-treated 2 h before induction with 5 μmol/L of AMPK inhibitor Compound C (Comp C, HY-13418, MCE, Monmouth, Junction, NJ, USA) or 5 μmol/L of STAT3 phosphorylation inhibitor Stattic (HY-13818, MCE, Monmouth, Junction, NJ, USA). The cells were subsequently divided into the following groups: NC, HG/PA, ALA, ALA + Comp C/ALA + Stattic. Following the 2-h inhibitor pretreatment, the culture medium was replaced with fresh medium containing HG/PA + ALA, and the culture was incubated for a further 24 h.

### 4.3. Measurement of Cell Viability and Cytotoxicity Assays

Cellular proliferation rates were quantified using Cell Counting Kit-8 (CK001; LABLEAD Trading Co., Ltd., Beijing, China), and cytotoxicity was evaluated according to lactate dehydrogenase activity (BC0658, Solarbio Science & Technology Co., Ltd., Beijing, China). The experimental procedures were strictly followed, and untreated cells cultured using standard conditions were used as negative controls.

### 4.4. Determination of MDA, GSH Levels

We homogenized H9C2 cells and collected the supernatant for testing. MDA levels were measured using a colorimetric lipid peroxidation detection kit (S0131S, Beyotime Biotechnology Co., Ltd., Shanghai, China). The principle of this test is based on the reaction of malondialdehyde (MDA) with thiobarbituric acid to produce a red product. The manufacturer’s instructions were closely followed to evaluate the MDA content in the H9C2 cell lysate, which reflects the degree of lipid peroxidation. The relative glutathione concentration in the cell lysate was measured using the Total Glutathione/Oxidized Glutathione detection kit (T-GSH/GSSG, E-BC-K097-M, Elabscience Biotechnology, Inc., Wuhan, China), following the manufacturer’s instructions for analysis.

### 4.5. Measurement of Mitochondrial Membrane (ΔΨm)

Mitochondrial membrane potential (ΔΨm) was measured using a JC-1 fluorescent probe (J22202, LABLEAD Trading Co., Ltd., Beijing, China). The treated H9C2 cells were incubated with JC-1 staining solution (37 °C, 30 min), washed twice with PBS, and resuspended in the culture medium. Fluorescence microscopy was used to detect JC-1 aggregates (ex/em 525/590 nm) and monomers (ex/em 485/530 nm). The red/green fluorescence intensity ratio served as an indicator of mitochondrial integrity.

### 4.6. Assessment of Reactive Oxygen Species

To measure intracellular reactive oxygen species (ROS) levels, we used the superoxide indicator 2′,7′-dichlorodihydrofluorescein diacetate (DCFH-DA, D6470, Solarbio Science & Technology Co., Ltd., Beijing, China). After treatment and culture, H9C2 cells were incubated with 10 μM of DCFH-DA and protected from light for 30 min at 37 °C. The cells were then washed twice with PBS and covered with DMEM. Fluorescent signals were recorded using a confocal fluorescence microscope, with emission set at 488 nm.

### 4.7. Animal Serum and Tissue Biochemical Assay

Mouse blood samples were obtained via orbital sinus puncture and allowed to clot overnight at 4 °C. Serum was isolated by centrifugation and subsequently analyzed for creatine kinase MB isoenzyme (CK-MB, ml107303, mlbio, Shanghai, China) and brain natriuretic peptide (BNP, ml037723, mlbio, Shanghai, China), using ELISA kits. Iron in the heart tissue was measured using serum iron ELISA kits purchased from Shanghai COIBO BIO (SI, CB10751-Mu, COIBO Biotechnology Co., Ltd., Shanghai, China). We used the following specific test kits to measure triglyceride (TG), total cholesterol (TC) and low-density lipoprotein cholesterol (LDL-C) levels: Blood sugar content detection kit (BC2495, Solarbio Science & Technology Co., Ltd., Beijing, China), triglyceride detection kit (A110-1-1, Nanjing Jiancheng Bioengineering Research Institute, Co., Ltd., Nanjing, China), total cholesterol detection kit (A111-1-1, Nanjing Jiancheng Bioengineering Research Institute) and low-density lipoprotein cholesterol detection kit (A113-1-1, Nanjing Jiancheng Bioengineering Research Institute, Co., Ltd., Nanjing, China). All experiments were conducted in accordance with the manufacturer’s instructions.

### 4.8. Fluorescence Staining

#### 4.8.1. FerroOrange Staining

H9C2 cells were exposed to ALA (40 μmol/L) for 24 h. Intracellular Fe^2+^ levels were assessed by staining with FerroOrange (1 μM, F374, Dojindo Laboratories, Kumamoto, Japan) for 30 min at 37 °C. The samples were examined under a confocal fluorescence microscope (Leica, Wetzlar, Germany) with excitation at 561 nm and emission recorded between 570 and 620 nm.

#### 4.8.2. Mito-SOX Staining

H9C2 cells were incubated with 40 μmol/L ALA for 24 h prior to mitochondrial superoxide detection. The cells were stained with 2 nM of Mito-SOX Red (40778ES50, Yeasen Biotechnology Co., Ltd., Shanghai, China) in PBS for 10 min at 37 °C under light-protected conditions. After washing, confocal microscopy images were acquired with 510 nm excitation and 580 nm emission wavelengths.

#### 4.8.3. BODIPY 581/591 C11 Staining

The lipid ROS levels in the cells were assessed by staining with BODIPY 581/591 C11 (Invitrogen, Carlsbad, CA, USA; Cat# D3861). The cells were incubated with the probe at a final concentration of 5 μM for 30 min (37 °C), followed by imaging using a confocal fluorescence microscope (Leica, Wetzlar, Germany).

#### 4.8.4. Immunofluorescence Staining

H9C2 cells were seeded on a glass-bottomed confocal culture dish. After treatment, the cells were fixed with 4% paraformaldehyde for 15 min at room temperature and subsequently permeabilized and blocked with 0.5% Triton-100 and 5% BSA in PBS for 1 h at room temperature. The cells were then incubated with primary antibodies diluted overnight at 4 °C. Following three PBS washes, the cells were incubated with a fluorescent secondary antibody for 1 h. After another round of triple PBS rinses, the nuclei were stained with DAPI Fluoromount-G^®^ (0100-20, Southern Biotech, Birmingham, AL, USA), with care taken to shield them from light. The H9C2 cells were then visualized under a confocal microscope (Leica, Wetzlar, Germany). For this experiment, primary antibodies directed against endogenous LC3B (1:100, A17424, ABclonal, Wuhan, China) and FTH1 (1:100, A19544, ABclonal, Wuhan, China) were employed.

### 4.9. RNA Sequencing and Analysis

RNA was extracted from the H9C2 cells using TRIzol reagent (BS259A, Biosharp, Beijing, China). The BGISEQ platform (BGI Genomics Co., Ltd., Shenzhen, China) was used to perform RNA sequencing and sequence quality control of cells in the NC, HG/PA, and ALA groups (three biological replicates per group). BGI’s Dr. Tom multi-omics data mining system was employed to generate heatmaps of transcriptomics data. The Kyoto Encyclopedia of Genes and Genomes (KEGG) database was queried for gene annotation using RNA sequencing data. We have uploaded all raw sequencing data generated in this study to the National Genomics Data Center (NGDC). The accession number for our dataset in the NGDC database is: https://ngdc.cncb.ac.cn/omix/preview/yTrji2h0 (accessed on 30 November 2025). 

### 4.10. Quantitative Real-Time PCR (RT-qPCR) Analysis

Total RNA was isolated with TRIzol reagent (BS259A, Biosharp Biotechnology Co., Ltd., Hefei, China) per the manufacturer’s protocol, and purity was verified using a NanoDrop 2000 (Thermo Fisher Scientific, Inc., Waltham, MA, USA). Reverse transcription of 1 μg of RNA was performed using Hifair III cDNA Synthesis SuperMix (11141ES60, Yeasen). qPCR amplification employed TransStart Top Green SuperMix (+Dye II) (AQ132-24, TransGen Biotech. Co., Ltd., Beijing, China) under established cycling conditions. Relative gene expression was calculated via the 2^(−ΔΔCt)^ method, normalized to β-actin. The primer sequences are listed in Table 1.

### 4.11. siRNA Transfection in H9C2

Rat-specific NCOA4 small interfering RNA (siRNA) and control-siRNA oligonucleotides were commercially synthesized by Sangon Biotech in Shanghai, China (see Table 2 for details). H9C2 cells were plated in fresh culture dishes for transfection using Lipofectamine 2000 (Cat# 40802ES03, YEASEN Biotechnology Co., Ltd., Shanghai, China) as the delivery vehicle. Following a 12-h incubation, the culture medium was replaced, and the cells were maintained for an additional 48 h. The knockdown efficiency of NCOA4 siRNA was subsequently verified by RT-qPCR and Western blotting.

### 4.12. Histological Examination

#### 4.12.1. Hematoxylin and Eosin (H&E) Staining

Mouse cardiac tissues were fixed in 4% paraformaldehyde, embedded in paraffin, and sectioned to a thickness of 5 μm. H&E (G1120, Solarbio, China) staining was used to observe structural characteristics. After staining, the slides were sealed with neutral resin, and images were collected using a Leica microscope (Leica, Wetzlar, Germany).

#### 4.12.2. Masson’s Trichrome Staining

Mouse cardiac tissues were fixed in 4% PFA, routinely dehydrated, paraffin-embedded, and sectioned to a thickness of 5 μm. Following standard dewaxing and rehydration, the sections were stained using a modified Masson’s trichrome kit (DC0032, LEAGENE Biotech Co., Ltd., Beijing, China) with the following chromatic characteristics: collagen fibers-blue; muscle fibers/cytoplasm-red; nuclei-blue, brown. This differential staining enabled a clear morphological distinction between collagen deposition (fibrosis) and the myocardial architecture.

#### 4.12.3. Detection of Iron Content

Cardiac tissue iron was identified with a Perls Prussian blue staining kit (Solarbio Science & Technology Co., Ltd., Beijing, China). In short, heart sections were heated at 60 °C for 60 min and rehydrated in distilled water. Next, the tissues were treated with the prepared working solution, a mix composed of equal parts potassium ferrocyanide and hydrochloric acid, for 3 min. Finally, the specimens were examined under a light microscope.

### 4.13. Protein Extraction and Western Blotting

Cardiac tissue and H9C2 cells were homogenized with precooled RIPA lysis buffer (R0010, Solarbio Science & Technology Co., Ltd., Beijing, China) containing 1 mM of the protease inhibitor (P0100, Solarbio, China) and 1 mM of the phosphatase inhibitor (P1260, Solarbio Science & Technology Co., Ltd., Beijing, China) for 20 min. Protein concentration was determined by BCA assay (B5001, Lablead Trading Co., Ltd., Beijing, China). Equal protein aliquots (30–40 μg) were resolved on 10–12% SDS-PAGE gels and electro transferred onto PVDF membranes. After blocking with 5% non-fat milk/TBST (2 h, RT), the membranes were probed with primary antibodies (4 °C, overnight). These included β-actin (ACTB, 81115-1-RR, Proteintech Group, Inc., Wuhan, China), p-AMPK (Thr172) (#2535, CST, Danvers, MA, USA), AMPK (#5831, CST, Danvers, MA, USA), p-STAT3 (Tyr 705) (#9145, CST, Danvers, MA, USA), STAT3 (#4904, CST, Danvers, MA, USA), NCOA4 (ab314553, abcam, Cambridge, MA, USA), FTH1 (#4393, CST, Danvers, MA, USA), SFXN1 (12296-1-AP, Proteintech Group, Inc., Wuhan, China), LC3B (#43566, CST, Danvers, MA, USA), ATG5 (#12994, CST, Danvers, MA, USA), P-ULK1 (#14202, CST, Danvers, MA, USA), SLC7A11 (A2413, ABclona, Wuhan, China), and GPX4 (67763-1-lg, Proteintech Group, Inc., Wuhan, China). The following secondary antibodies were employed: horseradish peroxidase (HRP)-conjugated goat anti-rabbit IgG (H+L) (S0101, Lablead Trading Co., Ltd., Beijing, China) and HRP-conjugated goat anti-mouse IgG (H+L) (S0100, Lablead Trading Co., Ltd., Beijing, China), both diluted at a ratio of 1:5000 and incubated for one hour at ambient temperature. Protein detection was performed using an enhanced chemiluminescence detection system(#170-5060, Bio-Rad, Hercules, CA, USA). Band intensity was quantified through densitometric analysis using ImageJ software, version 2 (NIH, Bethesda, MD, USA). For normalization purposes, β-actin (ACTB) served as the loading control to determine relative protein expression levels.

### 4.14. Echocardiography

Transthoracic echocardiography was performed in mice under 0.8% isoflurane anesthesia using a Vevo 2100 Imaging System (FUJIFILM VisualSonics, Inc., Toronto, ON, Canada) at the animal center of Capital Medical University (Beijing, China). E/A ratio, E’/A’ ratio, left ventricular ejection fraction (LVEF), and left ventricular fractional shortening (LVFS) were observed. Surface electrocardiogram and heart rates were also recorded.

### 4.15. Statistical Analysis

The data were processed with GraphPad Prism, version 10 (GraphPad Software Inc., La Jolla, CA, USA). The results are shown as the normalized meaning with the standard deviation (SD). For two-group comparisons, the unpaired Student’s *t*-test was used. When more than two groups were involved, one-way ANOVA followed by Tukey’s post hoc test determined significance. A *p*-value below 0.05 was deemed statistically significant.

## 5. Conclusions

In summary, our study demonstrated that ALA might alleviate DCM by inhibiting ferroptosis through two mechanisms: inhibiting NCOA4-mediated ferritinophagy and activating the SLC7A11/GSH/GPX4 antioxidant axis, both of which are regulated by the AMPK-STAT3 signaling pathway. These findings provide novel insight into the interplay between iron metabolism, redox balance, and metabolic signaling in DCM.

As with most studies, the design of the current study is subject to limitations. For instance, when designing animal experiments, we carefully reviewed multiple published studies in this field to establish animal models. We found that similar research subjects used male mice [65,66]. Therefore, male C57/6J mice were selected for our in vivo experiments, without considering the potential impact of sex differences on experimental outcomes. In future studies, we will incorporate mice of different genders into our research.

## Figures and Tables

**Table 1:** Primer sequences.

Gene Symbol	Species	Forward Primer (5′→3′)	Reverse Primer (3′→5′)
NCOA4	Rat	ACTTCAAGCACAGCATCC	TTCCAATAGCATAGGCAACT
FTH1	Rat	TGAGGTGTTGACTGACTTGGG	AAGCCCTGTGGCAAATCATC
SFXN1	Rat	GTCACGGTCATCACGATT	GCTTCTACGCTACTGTCAA
LC3B	Rat	CGTCCGAGAAGACCTTCAAA	CCTTGTATCGCTCTATAATCACTGG
ATG5	Rat	AGAAGAAGAGCCAGGTGATGAT	TGCTGATGTGAAGGAAGTTGTC
System XC	Rat	ATACGCTGAGTGTGGTTTGC	CTTCATCCACTTCCACAGCG
GPX4	Rat	AATTCGCAGCCAAGGACATC	GGCCAGGATTCGTAAACCAC

**Table 2:** siRNA sequence of NCOA4.

siRNA	Forward Primer (5′→3′)	Reverse Primer (3′→5′)
NCOA4-1	GCUGUUUCUCUCAGUCAAUTT	AUUCACUGAGAGAAACAGCTT
NCOA4-2	GCCCUACAAUGUCAAUGAUTT	AUCAUUCACAUUGUAGGGCTT
NCOA4-3	CCAUCAGGACACAUGUAAATT	UUUACAUGUGUCCUGAUGGTT
Negative control	UUCUCCGAACGUGUCACGUTT	ACGUGACACGUUCGGAGAATT

## Data Availability

Data is available and will be provided when it is required.
